# Communicating Information Regarding IBD Remission to Patients: Evidence From a Survey of Adult Patients in the United States

**DOI:** 10.1093/ibd/izae201

**Published:** 2024-08-28

**Authors:** Dallas W Wood, Katherine Treiman, Aileen Rivell, Welmoed K van Deen, Hilary Heyison, Mark C Mattar, Sydney Power, Alyssa Strauss, Gaurav Syal, Samantha Zullow, Orna G Ehrlich

**Affiliations:** RTI International, Research Triangle Park, NC, USA; RTI International, Research Triangle Park, NC, USA; RTI International, Research Triangle Park, NC, USA; Erasmus School of Health Policy and Management, Erasmus University Rotterdam, Rotterdam, The Netherlands; Weill Cornell Medical Center, New York Presbyterian, New York, NY, USA; Division of Gastroenterology, MedStar Georgetown University Hospital, Washington, DC, USA; Department of Medicine, University of North Carolina, Chapel Hill, NC, USA; Crohn’s & Colitis Foundation, New York, NY, USA; Division of Gastroenterology, University of California San Diego, La Jolla, CA, USA; Beth Israel Deaconess Medical Center, Harvard Medical School, Boston, MA, USA; Crohn’s & Colitis Foundation, New York, NY, USA

**Keywords:** inflammatory bowel disease, ulcerative colitis, Crohn’s disease, remission, patient-centered communication, communication barriers, education materials

## Abstract

**Background:**

Previous research suggests patients living with inflammatory bowel disease (IBD) understand IBD remission differently than healthcare professionals, which could influence patient expectations and clinical outcomes. We investigated 3 questions to better understand this: (1) How do patients currently understand remission; (2) Do patients currently face any barriers to communicating with their healthcare professional about remission; and (3) Can existing educational material be improved to help patients feel more prepared to discuss remission and treatment goals with their healthcare professional?

**Methods:**

We sent a web-based survey to adult patients with IBD in the United States. This survey included an educational experiment where patients were randomly assigned to 1 of 3 improved versions of existing educational material.

**Results:**

In total, 1495 patients with IBD completed the survey. The majority of patients (67%) agreed that remission is possible in IBD, but there was significant diversity in how they defined it with the most common being “my symptoms are reduced” (22%) and “I am no longer experiencing any symptoms” (14%). Patients reported being able to communicate openly with their healthcare professionals. Exposure to improved educational material did not have a statistically significant effect on patients’ feelings of preparedness for discussing different aspects of their care with their healthcare professionals.

**Conclusions:**

Our study confirms that patients tend to define remission in terms of resolving symptoms. We found little evidence of barriers preventing patients from discussing remission with their healthcare professionals. This suggests that educational material could be used to resolve this discrepancy in understanding.

**Key Messages**

**What is already known?**

• Previous research suggests that patients living with inflammatory bowel disease (IBD) understand IBD remission differently than healthcare professionals, which could influence patient expectations and clinical outcomes.

**What is new here?**

• We surveyed 1495 patients with IBD and confirmed that they tend to define remission in terms of resolving symptoms; we found little evidence of barriers preventing patients from discussing remission with their healthcare professionals.

**How can this study help patient care?**

• Our findings suggest that educational material could be used to resolve this discrepancy in understanding of remission between patients and healthcare professionals.

## Introduction

Approximately 2.4 million Americans are diagnosed with inflammatory bowel disease (IBD).^1^ IBD is an umbrella term used to describe disorders that cause chronic inflammation of the gastrointestinal tract. This inflammation can lead to symptoms such as abdominal pain, frequent bowel movements, and loose or watery stools. However, inflammation can be present even in the absence of symptoms. Although currently IBD cannot be cured, there are periods when the disease is not active. Remission is typically defined in 1 of 3 ways: (1) resolution of active IBD symptoms (ie, clinical remission),^2^ absence of disease activity observed during a colonoscopy (ie, endoscopic remission), or (3) absence of active inflammation at the tissue level (ie, histologic remission).^2^ Other remission levels, not discussed here, include biochemical and radiologic remission.

Previous research suggests that patients define remission as the resolution of active IBD symptoms, whereas healthcare professionals define remission based on objective tests (ie, endoscopic remission or histologic remission).^3^ Patients’ focus on symptom control when defining remission may reflect a difference in priorities, inadequate understanding of the disease and its treatment goals, or their difficulty observing disease activity when symptoms are not present.^4^

Recognition of the importance of minimizing disease activity at an early stage in IBD has led healthcare professionals to consider a “treat-to-target” (T2T) management approach.^5^ This collaborative approach between the healthcare professional and the patient involves identifying an appropriate target, selecting initial therapy according to the risk of disease progression, measuring baseline characteristics of disease, monitoring progress, and optimizing therapy to reach the agreed goal. For this collaborative approach to work, patients and healthcare professionals must use the same language so that expectations and communication align and share a common understanding of remission. One way to create a common understanding of remission is through patient education.

The goal of this study was to conduct a web-based survey of IBD patients to answer the following research questions: (1) How do patients currently understand remission, do they think remission is possible, and have they ever discussed remission with their healthcare professionals?; (2) Do patients currently face any barriers in communicating with their healthcare professional, and do they have preferences for how information should be received?; and (3) Can existing educational material be improved to help patients understand remission better and make them feel more prepared to discuss remission and treatment goals with their healthcare professional?

## Materials and Methods

Data for this study were collected using a web-based survey programmed and hosted by SurveyHealthcare Global (SHG; www.surveyhealthcareglobal.com), a healthcare market research firm. Specifically, SHG recruited US adults by e-mailing a survey link to their opt-in panel members. Individuals who were interested and clicked on the survey link were first asked to complete a brief screening questionnaire. This screening questionnaire asked for the patient’s age; whether the patient has been diagnosed with IBD or other conditions; and (in a follow-up question) whether the patient has been diagnosed with Crohn’s disease (CD), ulcerative colitis (UC), or “indeterminate colitis or IBD unclassified (IBD-U).” Respondents were eligible to complete the survey if they were adults (aged 18 or older) living in the United States who had been diagnosed with CD, UC, or IBD-U. Eligible respondents who completed the survey were compensated by SHG with reward points that could be redeemed later for various items.

The survey (see Supplementary Document S1) was informed by a series of 9 online focus groups that were conducted in 2021 with 43 adult patients diagnosed with IBD to understand how patients view remission in IBD. Patient groups were segmented by disease type (ie, CD or UC/IBD-U) and disease severity (mild, moderate, or severe). Patients were recruited for these focus groups by L&E Research, a marketing research firm. A moderator and co-moderator facilitated the online focus groups, using moderator guides. After introductions and an open-ended discussion of remission knowledge and what remission means to them, patients completed a remission factor ranking activity. Patient responses in the open-ended discussion on what remission means to them were used to create definitions of IBD remission that were included in 2 survey questions discussed below.

Before administering the survey, we pretested the survey instrument with 6 adult IBD patients. This pretest explored survey length and wording using in-person cognitive interviewing techniques.^6^ Patients participating in the pretest interview first completed the survey under timed conditions. Then, they reviewed their survey responses with the interviewer. During this portion of the interview, the patients were encouraged to “think aloud” and describe their thought process in answering each question. Based on the cognitive interview, we made several minor revisions to the survey to clarify question wording.

### Survey Instrument Contents

#### Patient characteristics

The first section of the survey obtained demographic information about the patients, including age, gender, ethnicity, race, educational background, and patients’ confidence level in completing medical forms independently. This section also asked whether individuals require assistance reading hospital materials, offering insights into their health literacy and support needs.

#### Patient experience with IBD

The second section of the survey focused on patients’ medical history, including the year of initial IBD diagnosis, symptoms they experienced over the past 6 months, whether they had any IBD-related surgeries, their current and past use of various IBD medications, and whether they had any recent emergency room visits or overnight hospital stays due to IBD.

#### Understanding of IBD remission

The third section of the survey asked patients to reflect on their understanding of remission in the context of IBD. First, patients were shown a list of statements describing IBD remission that was taken from the online-focused groups and were asked to select all the statements that describe what IBD remission means to them personally. Second, patients were shown the statements they had selected and were asked to select the single statement that best describes what IBD remission means to them personally. Next, patients were asked a series of “yes” or “no” questions on whether they believed IBD remission could be achieved, whether they believed someone could be considered in remission if they still had to take medication, whether they ever discussed remission with their healthcare professional, and whether they have ever been diagnosed as being in remission.

#### Patient communication preferences and experiences

The third section of the survey explores healthcare professionals’ role in providing patients with remission information. Specifically, patients were first asked to rate how important it was to discuss remission with their healthcare professional at different points in the course of their treatment (eg, when the patient was first diagnosed or after certain tests were completed). Patients were asked to rate the importance of these discussions on a scale from 1 (not at all important) to 5 (very important). Next, patients were asked to choose their preferred communication modes for receiving information about remission (ie, written material from their healthcare professional, information on a trusted website, an online video, from other patients with IBD, or other). Finally, patients were asked how well the healthcare professional responsible for treating their IBD has communicated with them over the past year. These questions were adapted from the validated Patient-Centered Communication in Cancer Care scale.^7^ The purpose of including these questions was to learn about patients’ perceived barriers in communicating with their healthcare professionals.

#### Educational experiment

The fourth section of the survey randomly assigned patients to read 1 of 3 versions of educational material explaining IBD remission and to answer a series of questions. The first version of the educational material included in the survey was the description of IBD remission currently available on the Crohn’s & Colitis Foundation (Foundation) website. This version of the educational material—the Control Version—represents how the Foundation currently describes IBD remission to patients. Next, we developed 2 alternative versions that attempt to improve on current practice: (1) the Updated Control Version and (2) the T2T Version. The exact text used for each version of the educational material is presented in Table 1. Patients were required to stay on this screen with the assigned educational material for at least 30 seconds to encourage them to read the text in full. After receiving the assigned educational material, patients were asked a series of questions. First, patients were asked to rate the readability of the information they received on a scale from 1 (very difficult to understand) to 5 (very easy to understand). We refer to this as the “ease of understanding” score. Next, patients were asked a question designed to capture how closely they read the educational material. Specifically, they were asked to select the long-term benefits of reaching endoscopic remission from a list of 5 possibilities (only 3 of which were correct). We refer to the number of benefits the patient correctly identified as the “correctness score.” Next, patients were asked to rate how prepared they felt to discuss different topics with their healthcare professional or to make medical decisions after reading the educational material on a scale from 1 (much less prepared) to 5 (much more prepared). We refer to this as the “preparedness score.” Finally, patients were asked how willing they were to pursue different treatments and tests to achieve remission on a scale from 1 (not at all willing) to 5 (very willing). We refer to this as the “willingness score.”

**Table 1.** Versions of educational material.

Version name	Version text
Control Version (current foundation website language)	Remission is a term that many people with IBD think of as “the absence of symptoms.” However, remission can take different forms, depending on what is being observed: *Symptom control/clinical remission* is the resolution of active IBD symptoms. This type of remission may come with a higher long-term risk of flaring and treatment failure compared with endoscopic or deep remission described below. This is because the treatments are focused on relieving your current symptoms, which can be helpful for patients to feel better. Other types of remission may target healing the damage and inflammation caused by your disease, as noted below. *Mucosal healing* (also known as endoscopic remission) refers to an absence of active disease seen during a colonoscopy (eg, no ulcers, no bleeding). Aiming for mucosal healing lowers your risk of complications such as strictures and fistulas. *Histologic remission* (also known as deep remission) occurs when no active inflammation is seen at the tissue level (when biopsies taken during a colonoscopy are examined under the microscope). Your healthcare professional(s) will prescribe treatments or in some cases recommend surgery to reach these remission goals.
**Updated** **Control** **Version** (Modifications: (1) dropped the use of terms such as “mucosal healing” and “deep remission” to match how endoscopic remission and histologic remission are described in recent Food and Drug Administration guidelines,^2^ added text to clarify how each form of remission differs from the others.)	The term “remission” is used to mean different things in IBD. *Clinical remission* means that IBD symptoms (eg, bloody stool, bowel urgency, pain, and fatigue) are under control. *Endoscopic remission* means there is no active disease seen during a colonoscopy (eg, no ulcers, no bleeding). *Histologic remission* means there is no active inflammation seen at the tissue level. Doctors determine whether a patient is in histologic remission by taking a biopsy during a colonoscopy and examining the tissue under a microscope. A patient who achieves clinical remission may feel better because their symptoms are under control. However, a patient may experience no symptoms and still have inflammation in their digestive system, which increases their risk of adverse outcomes in the future. Patients who reach histologic remission will have their symptoms under control and also have lower risk of flare-ups, lower risk of complications like strictures and fistulas, and lower risk of developing cancer of the colon or rectum in the future. You can work with your doctor or other healthcare professional to identify treatments that will help you reach your remission goals.
**T2T Version** (Modifications: defined symptom control as a short-term goal and remission as a long-term goal, where remission is defined as the absence of disease activity as observed through clinical tests.)	**Short-term treatment goal: Control of symptoms** The short-term treatment goal of most patients is to get their symptoms (eg, bloody stool, bowel urgency, pain, and fatigue) under control. However, a patient may experience no symptoms and still have inflammation in their digestive system, which increases their risk of adverse outcomes in the future. **Long-term treatment goal: Remission** The long-term goal of treatment is remission. When patients are in remission, their symptoms are under control, and there is no evidence of active disease (eg, ulcers or bleeding) and possibly no active inflammation at the tissue level. Doctors determine whether there is active disease by looking for ulcers or bleeding during a colonoscopy and by looking for signs of inflammation on scans. Doctors may determine whether there is active inflammation at the tissue level by taking a biopsy during a colonoscopy and examining the tissue under a microscope. Reaching remission has important benefits. Specifically, patients who reach remission will have their symptoms under control and also have lower risk of flare-ups, lower risk of complications like strictures and fistulas, and lower risk of developing cancer of the colon or rectum in the future. You can work with your doctor or other healthcare professional to identify treatments that will help you reach your treatment goals.

Abbreviations: IBD, inflammatory bowel disease.

### Quality Control

During data collection, we conducted a series of quality control checks to ensure that the final dataset would only include responses from real patients diagnosed with IBD and would exclude respondents who may not be answering the survey honestly in order to obtain the survey incentive. First, we excluded all respondents who completed the survey in less than 3 minutes because it did not seem feasible for an individual to complete the survey this quickly. Second, the screener question that asked respondents if they had been diagnosed with IBD included 5 other medical conditions to conceal our eligibility criteria from the respondent and minimize the possibility that some ineligible respondents could lie to be included in the survey. Third, at the end of the survey, we included an “attention check” question that asked respondents the type of IBD with which they had been diagnosed. If the answer to this question contradicted the first answer, their responses were excluded from the sample. Fourth, if the respondent’s answers seemed unlikely or infeasible to co-occur at existing prevalence their responses were excluded from the sample (see Supplementary Figure S1 for details). Our data collection partner (SHG) also had several checks and procedures to ensure that the same respondent could not complete the survey twice and that the respondent was a real person and not a bot answering questions randomly.

### Statistical Analysis

All results related to the first 2 research questions were summarized using descriptive statistics. To assess whether responses differed by IBD condition, we used a Chi-square test to determine whether proportions for all questions differed by IBD disease type subgroup. We also explored how patient definitions of IBD remission and communication preferences and experiences differed by IBD severity (patients experiencing IBD symptoms constantly or often over the past 6 months compared to other patients), duration (diagnosed for more than 10 years compared to 10 years or less), and patient age (patients over 40 years old compared to parents 40 years old or younger). We chose to divide the sample in this way because the divisions are both clinically meaningful and divide the sample approximately in half.

We answered the third research question by translating the outcome measures discussed above into continuous score variables. We then used an unpaired *t*-test to evaluate whether the values differ by subgroup. An unpaired *t*-test was used as the primary test to evaluate differences between subgroups because the *t*-test was expected to be robust to deviations from normality in large samples like those used in this survey.^8^ However, Winter and Dodou^9^ argue that the Wilcoxon rank-sum test can have power advantages when analyzing 5-point Likert items depending on the population distribution underlying the samples being evaluated. Therefore, as a robustness check, we also used a Wilcoxon rank-sum test to evaluate differences between groups. All analysis was conducted using Stata 15.^10^

### Ethical Considerations

RTI International’s Committee for the Protection of Human Subjects, which serves as RTI’s Institutional Review Board, reviewed and approved the study protocol before data collection. All patients provided consent to participate in the study.

## Results

### Patient Characteristics and Experience With IBD

Data were collected in several waves over a 9-week period from October to December 2023. After performing the data quality checks described above, 1495 patients were included in the final sample used for our analysis (see Supplementary Figure S1 for details). The median response time for the survey was 10.1 minutes (IQR 7.5-14.2). Descriptive statistics of the samples we collected are provided in Table 2 by IBD disease type. These data are also summarized by disease severity, duration, and patient age in Supplementary Tables S2, S6, and S10, respectively. The majority of the 1495 patients were younger than 45 years old (57.5%), female (63.0%), White (75.2%), and college educated (82.1%).

**Table 2.** Patient characteristics.

Characteristic	IBD condition			
	Crohn’s disease (*n* = 887)	Ulcerative colitis (*n* = 545)	Indeterminate colitis (*n* = 63)	Total (*n* = 1495)
Age, % (*P* < .01)				
18-24	6.0	4.0	6.4	5.3
25-44	56.8	44.4	54.0	52.2
45-64	25.7	35.4	28.6	29.4
65+	11.5	16.2	11.1	13.2
Gender, % (*P* = .04)				
Female	59.9	67.9	65.1	63.0
Male	39.4	31.7	34.9	36.4
Non-binary/Other	0.8	0.4	0.0	0.6
Race/ethnicity, % (*P* = .74)				
White, non-Hispanic	74.0	77.3	74.6	75.2
Black, non-Hispanic	5.6	6.1	6.4	5.8
Asian, non-Hispanic	1.2	0.9	1.6	1.1
Other, non-Hispanic	4.3	3.3	7.9	4.1
Hispanic	13.9	11.6	9.5	12.8
Prefer not to answer	1.0	0.9	0.0	0.9
Education, % (*P* = .59)				
High school graduate or less	18.8	17.1	12.7	17.9
Some college or 2-year degree	33.8	36.9	36.5	35.1
4-year degree or more	47.4	45.9	50.8	47.0
Prefer not to answer	0.0	0.2	0.0	0.1
Years since patient’s IBD was first diagnosed, % (*P* < .01)				
1-2 years	16.6	11.7	36.5	15.7
3-5 years	21.4	21.3	23.8	21.5
6-10 years	17.8	18.5	7.9	17.7
More than 10 years	44.2	48.4	31.8	45.2
Healthcare professional treating patient’s IBD, % (*P* = .38)				
Gastroenterologist	79.0	79.1	68.3	78.6
Primary care doctor	17.7	16.5	28.6	17.7
Nurse practitioner or physician	1.2	1.8	1.6	1.5
Fellow or resident	0.6	0.2	0.0	0.4
Other specialist	0.8	1.5	0.0	1.0
Not sure or don’t remember	0.7	0.9	1.6	0.8
Total	100	100	100	100

Abbreviation: IBD, inflammatory bowel disease.

*P*-value obtained from the Chi-square test for the differences in responses across IBD subgroups.

### Patient Experience With IBD

Table 3 summarizes patient experiences with IBD by IBD disease type. Approximately half of patients (51%) identified their disease as being constantly or often active over the past 6 months. The most common symptom across all IBD conditions was abdominal pain or cramps. We found that 22% of patients had surgery for IBD—specifically, 3% had a colectomy and 4% had an ostomy. In the 6 months before taking the survey, 30% of patients visited an emergency room for IBD, and 25% had an overnight stay in a hospital. Active medication use by patients included biologic (29%), corticosteroids (28%), 5-ASAs (23%), immunomodulators (15%), and a targeted synthetic small molecule (7%).

**Table 3.** Experiences with IBD and IBD treatment.

Experience	IBD condition			
	Crohn’s disease (*n* = 887)	Ulcerative colitis (*n* = 545)	Indeterminate colitis (*n* = 63)	Total (*n* = 1495)
Severity, % (*P* < .01)				
Constantly active	22.0	16.7	30.2	20.4
Often active	32.7	25.9	34.9	30.3
Sometimes active	24.2	23.9	19.1	23.9
Occasionally active	10.4	13.6	6.4	11.4
Rarely active	6.1	11.4	1.6	7.8
No symptoms	4.6	8.6	7.9	6.2
Top 5 IBD symptoms patient experienced in past 6 months, %				
Abdominal pain or cramps	72.7	71.6	82.5	72.7
Frequent bowel movements	64.6	59.5	58.7	62.5
Bowel urgency	60.0	61.8	61.9	60.7
Fatigue or feeling very tired	59.6	59.8	60.3	59.7
Loose or watery stools	60.1	58.4	57.1	59.3
Experiences with IBD, %				
Had surgery for IBD (*P* < .01)	29.5	11.6	14.3	22.3
Currently has an ostomy (*P* < .01)	4.8	2.9	1.6	4.0
Has had a colectomy (*P* < .01)	3.5	2.8	4.8	3.3
Visited ER for IBD in past 6 months (*P* < .01)	33.6	23.7	34.9	30.0
Overnight hospital stay for IBD in past 6 months (*P* < .01)	27.2	19.3	36.5	24.7
Medication patient is currently taking, %				
Biologics (*P* < .01)	34.8	21.5	14.3	29.1
Corticosteroids (*P* < .01)	30.7	24.8	28.6	28.4
Aminosalicylates (5-ASAs) (*P* < .01)	19.5	29.4	20.6	23.1
Immunomodulator (*P* < .01)	17.5	13.0	7.9	15.5
Antibiotics (*P* < .01)	16.1	9.2	27.0	14.1
Targeted synthetic small molecules (*P* < .01)	7.3	6.1	6.4	6.8

Abbreviation: IBD, inflammatory bowel disease.

*P*-value obtained from Chi-square test for the differences in responses across IBD subgroups.

### Understanding of and Experience With IBD Remission

Table 4 presents results for which statements patients believed best described IBD remission by IBD disease type. Patients tended to define remission in terms of symptoms and quality of life. The most common definition of remission was “my symptoms are reduced” (22%). This was true across IBD type, severity, duration, and patient age (Supplementary Tables S3, S7, and S11, respectively). Other common definitions of remissions were based on symptom control such as, “I am no longer experiencing any symptoms” (14% of full sample), and “I can carry out everyday activities” (13% of full sample). However, we did identify some differences across subgroups. For example, in terms of IBD type, Crohn’s patients were most likely to define remission as “my disease is no longer pressing or worsening” (14%); and indeterminate colitis patients were most likely to define remission as “My doctor or other healthcare professional tells me that my colonoscopy, labs, or scan results show no signs of disease” (14%). Of those who selected “my disease is no longer pressing or worsening,” the top concern with disease progression was having to undergo surgery or another surgery (40%) (see Supplementary Table S1 for details).

**Table 4.** Statements patients identified as best description of what remission related to IBD means to them.

Statement	IBD condition			
	Crohn’s disease (*n* = 887)	Ulcerative colitis (*n* = 545)	Indeterminate colitis (*n* = 63)	Total (*n* = 1495)
My symptoms are reduced (eg, less pain, bowel urgency, fatigue).	21.5%	22.2%	20.6%	21.7%
I am no longer experiencing any symptoms (eg, no bowel urgency, pain, fatigue).	12.3%	16.9%	4.8%	13.7%
I can carry out everyday activities (eg, work, social activities).	12.6%	13.6%	7.9%	12.8%
My disease is no longer progressing or worsening.	14.0%	7.9%	6.4%	11.4%
I feel so well that I no longer need to take any medications for my disease.	9.2%	11.0%	11.1%	10.0%
My doctor or other healthcare professional tells me that my colonoscopy, labs, or scan results show no signs of disease.	9.8%	9.2%	14.3%	9.8%
I can reduce the dose or frequency of medications	7.8%	5.7%	9.5%	7.1%
I feel the way I used to feel before I got sick.	4.4%	6.2%	11.1%	5.4%
My doctor or other healthcare professional tells me that my colon/intestine is healed.	1.4%	2.2%	6.4%	1.9%
Not sure	4.4%	2.4%	3.2%	3.6%
Other	2.6%	2.8%	4.8%	2.7%
Total	100.0%	100.0%	100.0%	100.0%

Abbreviation: IBD, inflammatory bowel disease.

Test for differences in responses across IBD subgroups using Chi-square test (*P* < 0.01).

Table 5 presents results for the patient understanding of IBD remission by IBD disease type. About 67% of patients agreed that remission was possible with IBD, 62% reported that they had discussed remission with their healthcare professional, 49% agreed that remission could involve taking medication, and 38% said their healthcare professional had told them that they were in remission. There were statistically significant differences in responses based on IBD severity, duration, and patient age (Supplementary Tables S4, S8, and S13). Several of these differences were relatively small (ie, smaller than 5 percentage points). However, some differences were larger and worth noting here. Patients with more severe IBD were less likely to believe IBD remission was possible, more likely to have discussed remission with a doctor, and less likely to have ever been told they were in remission than patients with less severe IBD. Patients diagnosed with IBD for more than 10 years were less likely to consider themselves in remission if still on medication and more likely to have been told they were in remission. Patients over 40 years old were less likely to believe IBD remission is possible, less likely to consider themselves in remission if still on medication, less likely to have discussed IBD with a doctor, and less likely to have ever been told they were in remission than patients 40 years old or younger.

**Table 5.** Patient understanding of inflammatory bowel disease remission.

Agree with statement	IBD condition			
	Crohn’s disease (*n* = 887)	Ulcerative colitis (*n* = 545)	Indeterminate colitis (*n* = 63)	Total (*n* = 1495)
IBD remission is possible, % (*P* < .01)	67.9	66.1	54.0	66.6
Would consider self to be in remission if still on medication, % (*P* = .02)	52.5	45.3	33.3	49.1
Ever discussed remission with doctor, % (*P* < .01)	65.8	55.8	49.2	61.5
Ever been told was in remission, % (*P* = .01)	40.8	35.4	27.0	38.3

Abbreviation: IBD, inflammatory bowel disease.

P-values obtained from Chi-square test for the differences in responses across IBD subgroups.

### Patient Communication Preferences and Experiences

Table 6 summarizes patient communication preferences and experiences by IBD disease type. Patients preferred to receive information about IBD remission through written material from their healthcare professionals (78%) or trustworthy online websites (74%) compared to online video or talking with other patients. This was true across IBD type, severity, duration, and patient age (Supplementary Tables S5, S9, and S14, respectively). Similarly, more than 60% of patients indicated that it was somewhat important or very important to discuss remission at every point in time considered. This was also true across IBD type, severity, duration, and patient age (Supplementary Tables S5, S9, and S14, respectively). In terms of rating their previous experience communicating with the healthcare professional responsible for their IBD case, 79% of patients said their healthcare professional always or often has honest communication with them, 73% said their healthcare professionals did a great deal or a lot to make them feel comfortable asking questions, 66% said their healthcare professional always or often made sure they understood the steps in their care, 62% said their healthcare professionals did a great deal or a lot provide information to help them make medical decisions, 53% said their healthcare professionals did outstanding or very well helping them deal with uncertainties about IBD, and 52% said their healthcare professionals did outstanding or very well when talking with them about how to cope with stress and other feelings related to living with IBD.

**Table 6.** Patient communication preferences and experiences.

Communication preference/experience	IBD condition			
	Crohn’s disease (*n* = 887)	Ulcerative colitis (*n* = 545)	Indeterminate colitis (*n* = 63)	Total (*n* = 1495)
Percentage of patients selecting a form of communication as 1 of 2 most preferred ways of receiving information on remission				
Written material from my doctor that I could take home with me, % (*P* = .34)	77.6	80.4	74.6	78.5
Information on a website that I can trust, % (*P* = .13)	72.4	77.1	71.4	74.1
A video I can watch online, % (*P* = .21)	24.9	21.8	30.2	24.0
From other patients with IBD, % (*P* = .24)	23.6	19.8	23.8	22.2
Other, % (*P* = .36)	1.6	0.9	0.0	1.27
Percentage of patients saying it is important or very important to discuss remission with your healthcare professional at each point in time				
When I am first told I have IBD, % (*P* = .64)	67.3	68.1	73.0	67.8
After my symptoms are under control, % (*P* = .67)	78.0	76.3	74.6	77.3
During each visit with my doctor or healthcare professional, % (*P* = .57)	61.6	62.4	68.3	62.1
During flare-ups, % (*P* = .43)	62.0	65.3	65.1	63.3
After a colonoscopy, % (*P* = .02)	75.2	81.3	74.6	77.4
After getting test or lab results, % (*P* = .19)	73.7	77.3	81.0	75.3
Percentage of patients agreeing with statement about communicating with healthcare professional over past year				
Makes me feel comfortable, % (*P* = .22)	74.5	72.4	65.1	73.4
Communicates honestly, % (*P* = .16)	79.6	77.7	69.8	78.5
Provides information, % (*P* = .01)	65.4	57.4	60.3	62.3
Helps me cope with stress from IBD, % (*P* = .04)	54.5	47.5	52.4	51.8
Helps me understand steps involved in care, % (*P* = .61)	66.3	66.4	60.3	66.1
Helps me deal with uncertainties, % (*P* < .01)	55.9	48.4	42.9	52.6

Abbreviation: IBD, inflammatory bowel disease.

*P*-values obtained from Chi-square test for the differences in responses across IBD subgroups.

### Education Experiment

Table 7 presents the results of the education experiment. Between-group comparisons revealed that patients assigned to the Updated Control Version of the educational material identified fewer benefits of endoscopic remission correctly (mean = 1.73; 95% confidence interval [CI], 1.64-1.81) than patients assigned to the Control Version (mean = 1.89; 95% CI, 1.81-1.97). However, Updated Control Version patients rated the readability of the educational material (mean = 4.54; 95% CI, 4.48-4.60) higher than Control Version patients (mean = 4.39; 95% CI, 4.32-4.46). Between-group comparisons also reveal that patients assigned to the T2T Version correctly identified more benefits of remission (mean = 2.31; 95% CI, 2.23-2.39) than patients assigned to the Control Version (mean = 1.89; 95% CI, 1.81-1.97) or the Updated Control Version. Patients assigned to the T2T Version also rated the readability of the educational material (mean = 4.70; 95% CI, 4.65-4.75) higher than patients assigned to the Control Version (mean = 4.39; 95% CI. 4.32-4.46) or Updated Control Version. The Updated Control and T2T Versions did not have a statistically significant effect on patients’ feelings of preparedness for discussing different aspects of their care with their healthcare professionals or patients’ willingness to undergo various treatments and tests to achieve remission. The statistical significance of the between-group comparisons above does not change when they are evaluated using the Wilcoxon rank-sum test.

**Table 7.** Exploring effect of educational material on outcome measures.

Score description	Control version (*n* = 500)	Updated control version (*n* = 497)	T2T version (*n* = 498)	Updated control version vs control	T2T version vs control	Updated control version vs T2T version
	Mean correctness score [95% confidence intervals]					
Number of correctly identified benefits	1.89 [1.81-1.97]	1.73 [1.64-1.81]	2.31 [2.23-2.39]	−0.17 [−0.28 to −0.05]	0.42 [0.31-0.52]	−0.58 [−0.69 to −0.47]
	Mean ease of understanding score [95% confidence intervals]					
Ease of understanding	4.39 [4.32-4.46]	4.54 [4.48-4.60]	4.70 [4.65-4.75]	0.15 [0.06-0.25]	0.31 [0.22-0.40]	−0.16 [−0.23 to −0.08]
	Mean preparedness scores [95% confidence intervals]					
Discuss remission with their doctor	4.19 [4.12-4.26]	4.15 [4.08-4.22]	4.16 [4.09-4.23]	0.04 [−0.14-0.06]	−0.03 [−0.13-0.07]	−0.01 [−0.1-0.1]
Discuss treatment goal with their doctor	4.17 [4.10-4.24]	4.19 [4.13-4.26]	4.12 [4.05-4.19]	0.02 [−0.08-0.12]	−0.05 [−0.15-0.05]	0.07 [−0.03-0.17]
Make decisions about their treatment	4.18 [4.11-4.25]	4.16 [4.08-4.23]	4.13 [4.06-4.20]	−0.02 [−0.13-0.08]	−0.05 [−0.15-0.5]	0.03[−0.07-0.13]
	Mean willingness scores [95% confidence intervals]					
Continue using medications	4.29 [4.21-4.38]	4.28 [4.20-4.36]	4.34 [4.26-4.42]	−0.01 [−0.13-0.11]	0.05 [−0.07-0.17]	−0.06 [−0.18-0.06]
Have an additional colonoscopy	3.86 [3.76-3.96]	3.82 [3.72-3.92]	3.95 [3.86-4.05]	−0.04 [−0.18-0.11]	0.09 [−0.05-0.23]	−0.13 [−0.27-0.01]
Have more frequent labs and other tests	4.16 [4.08-4.24]	4.21 [4.12-4.29]	4.18 [4.09-4.26]	0.05 [−0.07-0.17]	0.15 [−0.10-0.13]	0.03 [−0.09-0.15]

Abbreviation: T2T, treat-to-target.

## Discussion

Previous research has suggested that patients and healthcare professionals differ in their understanding of remission.^3^ Specifically, patients typically define remission in terms of the absence of symptoms, whereas healthcare professionals define remission in terms of objective test results. Differences in understanding of remission could complicate treatment approaches like T2T, which rely on collaboration between the healthcare professional and the patient. This study contributes to the literature in 2 ways. First, we conducted one of the first large sample surveys to understand how patients currently define remission and investigate patient experiences and preferences for communicating with their healthcare professional. Second, this is the first study to assess whether patient educational material can be used to help patients understand IBD remission better, feel more prepared to discuss treatment options with their healthcare professional, and be more willing to undergo different treatments and tests to achieve remission.

This study addressed how patients currently understand remission: Nearly two-thirds of patients agreed that remission is possible in IBD and had discussed IBD remission with their healthcare professional, and more than one-third of patients had been told by their healthcare professional at some point that they were in remission. However, about half of patients believed remission would not entail taking any medication. In addition, we found significant variation in how patients defined remission, but the most common definitions of remission related to the absence or reduced appearance of symptoms. These results did not differ by disease type, severity, duration, or patient’s age. Previously, Rubin et al.^11^ and Schreiber et al.^12^ conducted patient surveys that asked patients how they defined remission. However, both studies exclusively focused on whether patients define remission to mean the complete absence of symptoms as opposed to experiencing some symptoms. Neither study asked whether patients consider the results of clinical tests to be a better way to define remission. The only study that explored whether patients primarily define remission in terms of symptoms or clinical tests was a 2021 study by Rubin et al.,^3^ which found that the most common way patients defined remission was as the resolution of IBD symptoms. It is important to emphasize that, even though our study attempted to improve on the 2021 Rubin et al. study^3^ by offering more ways to define IBD remission, both studies found similar results. However, our results differ from Rubin et al.’s results in a few ways. Specifically, a greater proportion of patients responding to Rubin et al.’s survey had previously discussed IBD remission with a healthcare professional (78% vs 61.5% in our survey). This difference may be because part of Rubin et al.’s sample was recruited through patient advocacy groups and they commented that patients who interact with these groups may be more involved with their own care than patients who do not.

We did not find evidence of widespread barriers to communication between patients and healthcare professionals. We found that two-thirds or more of patients felt comfortable discussing their treatment with their healthcare professional. Similarly, we also found that more than two-thirds of patients felt that their healthcare professional communicated honestly with them, provided a significant amount of information on their care, and helped them understand the steps involved with their care. These results are based on questions from the validated Patient-Centered Communication in Cancer Care scale.^7^ This is the first study to use this scale in a survey of IBD patients, so no direct comparisons can be found in the literature. However, our results were consistent with individual questions from Rubin et al. that targeted similar aspects of patient-physician communication.^3^ Regarding patient preferences for receiving information on IBD remission, we found that a clear majority of patients preferred receiving written information from their healthcare professional or from a trusted website.

Evaluating whether existing educational material on IBD remission could be improved to help patients understand remission better and feel more prepared to discuss remission and treatment goals with their healthcare professionals is a novel contribution of our research to the literature. As Husain and Triadafilopoulos^13^ note, education is one of the most significant interventions for improving the lives of patients with IBD, but limited data are available on which communication methods are most effective for healthcare professionals. We found that educational material that discussed remission in terms of short- and long-term treatment goals and focused on a single definition of remission performed the best in patient-reported ease of understanding and patient comprehension of educational material contents. None of the educational material we considered did significantly better or worse than the Control Version in terms of making patients feel more prepared to discuss remission with their healthcare professional or making patients more willing to undergo a particular treatment or test.

We recognize some limitations of our study. First, we conducted this survey using a convenience sample drawn from an opt-in online panel where the patients’ IBD diagnosis and other characteristics were self-reported. As a result, it is possible that patients did not answer questions truthfully and that our sample is not representative of the true population of patients with IBD. To mitigate the threat of inaccurate responses, we included a number of quality checks to identify and remove respondents who may have been answering questions falsely. To explore the possibility that our sample is not representative, we investigated how the characteristics of our sample differed from the characteristics of IBD patients at large. Overall, we found the demographic characteristics of our sample were broadly consistent with the demographic characteristics of the IBD population in terms of the prevalence of each IBD condition and the sex and ethnicity of patients.^14^ However, we did identify some differences. In particular, 50.7% of our sample had “severe” IBD where they experience IBD symptoms constantly or often. This is also reflected in the fact that 30% of our sample visited an emergency room due to IBD, 24.7% had to stay overnight in a hospital due to IBD, and 22% of patients with UC were currently taking biologics. Based on the existing literature, these prevalences seem potentially higher than what might be expected.^15–17^ To understand this discrepancy, it is essential to note that SHG often works with pharmaceutical firms. As a result, surveys SHG administers for these clients could often relate to patients taking biologics. A greater frequency of survey opportunities for these patients could encourage their persistence in the panel and lead to an over-representation of these patients relative to the IBD patient population as a whole. To understand its potential impact on the study results, we explored whether patient understanding of IBD remission and communication differences differ by IBD severity within our sample. Overall, we found that, regardless of IBD severity, patients most commonly define remission in terms of relief from symptoms and preferred receiving written information from their healthcare professional or from a trusted website.

A related limitation is that individuals who join opt-in panels may be systematically different from individuals who do not, which creates the possibility of selection bias. Although selection bias can be difficult to address, some research suggests that convenience samples from opt-in panels can yield findings for experiments that are similar to those based on probability-based samples, even when the demographic characteristics of the samples differ substantially.^18^

Another limitation is that, when evaluating the effectiveness of educational material, we relied on hypothetical questions regarding whether patients would feel more prepared to talk with their healthcare professional or would be more willing to undergo various treatment options to achieve remission. It is possible that patients’ answers would differ if they actually had to talk with their healthcare professional about remission or if they had to consider different treatment options. We hope future research efforts can address these limitations in subsequent studies.

## Conclusions

This study suggests a significant discrepancy in how patients and healthcare professionals define remission, which could affect patient expectations and clinical outcomes. We found little evidence of barriers preventing patients from discussing remission in more detail with their healthcare professionals. This suggests that educational materials could be used to resolve this discrepancy in understanding. Patients tend to prefer receiving educational materials either from their healthcare professional or from a trusted website. In our educational experiment, a version of the educational material that was designed to discuss remission in terms of short- and long-term treatment goals and that focused on a single definition of remission performed the best in terms of patient-reported ease of understanding and patient comprehension of educational material contents. This can leave patients feeling prepared to talk with their healthcare professional.

## Supplementary data

Supplementary data is available at *Inflammatory Bowel Diseases* online.

izae201_suppl_Supplementary_Document_S1

izae201_suppl_Supplementary_Material
