# Supplementary material for: Communicating Information Regarding IBD Remission to Patients: Evidence From a Survey of Adult Patients in the United States
Source: Inflamm Bowel Dis. 2024 Aug 28;31(6):1605–15. doi: 10.1093/ibd/izae201 (PMC12166295; doi:10.1093/ibd/izae201)
Supplement: izae201_suppl_Supplementary_Document_S1 [file izae201_suppl_supplementary_document_s1.docx]

**SUPPLEMENTAL MATERIALS**

Communicating Information Regarding IBD Remission to Patients: Evidence from a Survey of Adult Patients in the United States

Dallas W. Wood, Katherine Treiman, Aileen Rivell, Welmoed K. van Deen, Hilary Heyison,Mark C. Mattar, Sydney Power, Alyssa Strauss, Gaurav Syal, Samantha Zullow, Orna G. Ehrlich

**Supplemental Tables**

**Table S1.** Flow Diagram for Survey Response Dataset Construction

**Supplemental Figures**

**Figure S1.** Physician-Reported Remission and Loss of Response With Current Treatment Options

**Supplemental Document**

**Document S1.** Survey Instrument

**Supplemental Table S1.** Patient top concern with disease progression

|  | **Crohn's Disease with surgery (n=111)** | **Crohn's Disease with no surgery (n=253)** | **All Crohn's Disease Patients (n=364)** |
| --- | --- | --- | --- |
| Having to undergo surgery / Having to undergo another surgery | 47.8% | 37.2% | 40.4% |
| My symptoms returning | 18.0% | 16.2% | 16.8% |
| Having to increase treatment dosage | 0.0% | 2.8% | 1.9% |
| Having to take a new treatment | 2.7% | 3.6% | 3.3% |
| Greater risk of cancer in the future | 14.4% | 18.2% | 17.0% |
| Risk of long-term side effects from stronger/different medications | 12.6% | 15.8% | 14.8% |
| Losing response to current treatment | 2.7% | 4.7% | 4.1% |
| Other | 1.8% | 1.6% | 1.6% |
| **Total** | **100.0%** | **100.0%** | **100.0%** |

Note: Note: Data for ulcerative colitis patients excluded due to low response rates for this question.

**Supplemental Figure S1. Flow Diagram for Survey Response Dataset Construction**

**<<Insert Figure S1 Here>>**

^a^ We excluded 492 respondents for failing at least one quality check. The number of respondents excluded due to each quality check does not add up to 492 because respondents could fail more than one quality check. We excluded 315 respondents who had both a colectomy and an ostomy and 215 respondents who were currently taking a biologic and targeted small molecule drug. Although it is possible for a patient to have both a colectomy and an ostomy or to take both a biologic and targeted small molecule drug, it is very unlikely. Therefore, we excluded this relatively large number of respondents to avoid compromising data quality.

**ALT TEXT FOR FIGURE S1.** A flow chart that shows how the analytical sample was created. Specifically, 1,987 patients completed the survey. Of these 1,987 patients, 492 patients were excluded for failing at least one quality check described above. Specifically, 16 patients were excluded for completing the survey in less than 3 minutes, 158 were excluded because they failed the attention check question, 315 were excluded because they said they had both a colectomy and an ostomy, and 215 were excluded because they indicated they were currently taking both a biologic and a targeted synthetic small molecule at the same time. Note that these numbers do not add up to 492 because patients could fail more than one quality check. This resulted in 1,495 patients being included in the final sample used for our analysis.

In addition to the quality checks reported in Figure S1, we also identified 22 respondents that indicated they had all six medical conditions included in the screener question used to determine if the patient had been diagnosed with IBD. Although it seems unlikely that a single individual would have all six medical conditions, we found that our results did not change whether we included these patients or not. Therefore, we chose to include them in the final sample.

Supplemental Document S1. Survey Instrument

[Section A. SCREENER]

[MANDATORY QUESTION]

[NUMERICAL FIELD; INTEGER RANGE 0 – 100]

A1. What is your current age?

[Text Entry]

[IF A1 < 18, TERMINATE]

[MANDATORY QUESTION]

[radio; single punch (Randomize order of response options)]

A2. Have you ever been told by a doctor or other healthcare professional that you had any of the following digestive diseases or conditions? Select all that apply.

1 Inflammatory bowel disease (IBD) such as Crohn’s disease or ulcerative colitis

2 Celiac disease

3 Gastroesophageal reflux disease (Reflux)

4 Eosinophilic esophagitis

5 Gastroparesis

6 Irritable bowel syndrolegme (IBS)

7 Other (specify)

8 I have not been told by a doctor or other healthcare professional that I had any of these conditions

[IF (A2 != 1 ‘Inflammatory Bowel Disease (IBD)’), set EFLAG = 0 ‘Ineligible’]

[IF A2 = 1]

[MANDATORY QUESTION]

[radio; single punch]

A3. Which of the following inflammatory bowel disease (IBD) conditions were you most recently told by a doctor or other healthcare professional that you had?

1 Crohn’s disease (CD)

2 Ulcerative colitis (UC)

3 Indeterminate Colitis or Unclassified IBD (IBD-U)

5 Other (please specify)

6 Prefer not to answer

[IF (A3 = 5 or 6), set EFLAG = 0 ‘Ineligible’]

[IF A2 = 1]

[MANDATORY QUESTION]

[radio; single punch]

A4. In the last 6 months, my IBD has been

1 Constantly active, giving me symptoms every day.

2 Often active, giving me symptoms most days

3 Sometimes active, giving me symptoms on some days (for instance 1-2 days per week)

4 Occasionally active, giving me symptoms 1-2 days per month

5 Rarely active, giving me symptoms on a few days in the past 6 months

6 I was well in the past 6 months and had no symptoms

[IF (A4 = 1 or 2), set SEVERITY_FLAG to ‘severe’

If (A4 = 3 or 4), set SEVERITY_FLAG to ‘moderate’
If (A4 = 5), set SEVERITY_FLAG to ‘mild’]

If (A4 = 6), set SEVERITY_FLAG to ‘remission]

[IF A2 = 1]

[MANDATORY QUESTION]

[radio; single punch]

A5. What type of health care professional do you see primarily for your IBD care?

1 Primary care doctor (e.g., internist, family medicine doctor)

2 Gastroenterologist (a doctor who specializes in the digestive system)

3 Nurse Practitioner or Physician Assistant

4 Fellow or resident (i.e., physician undergoing period of medical training)

5 Other specialist (please specify)

6 Not sure or don’t remember

[MANDATORY QUESTION]

[radio; SINGLE punch]

A6. With which of the following gender categories do you most identify? Please select one.

1 Female

2 Male

3 Non-binary

4 Other

5 Prefer not to answer

[MANDATORY QUESTION]

[radio; single punch]

A7. What is your ethnicity? Please select one.

1 Hispanic or Latino

2 Not Hispanic or Latino

3 Prefer not to answer

[MANDATORY QUESTION]

[CHECKBOX; MULTI-PUNCH]

A8. What is your race? Select all that apply.

A8_1. Asian (East Asian, South Asian, Southeast Asian)

A8_2. Black or African American

A8_3. Middle Eastern or North African

A8_4. Native American

A8_5. Native Hawaiian or Other Pacific Islander

A8_6. White

A8_7. Other

A8_8. Prefer not to disclose

[MANDATORY QUESTION]

[radio; single punch]

A9. What is the highest degree or level of school you have completed?

1 Less than high school

2 High school graduate or GED

3 Some college but no degree

4 Associate degree (for example: AA, AS)

5 Bachelor’s degree (for example: BA, BS)

6 Postgraduate or advanced professional degree (for example: MA, MS, PhD, MD, DDS, JD)

7 Prefer not to answer

[MANDATORY QUESTION]

[radio; single punch]

A10. How confident are you in filling out medical forms by yourself?

1 Not at all

2 A little bit

3 Somewhat

4 Quite a bit

5 Extremely

[MANDATORY QUESTION]

[radio; single punch]

A11. How often do you have someone help you read hospital materials?

1 All the time

2 Most of the time

3 Some of the time

4 A little bit of the time

5 None of the time

[MANDATORY QUESTION]

[radio; single punch]

A12. How often do you have problems learning about your medical condition because of difficulty understanding written information?

1 All of the time

2 Most of the time

3 Some of the time

4 A little bit of the time

5 None of the time

[----------------------------------------New screen----------------------------------------]

[DISPLAY ‘Thank and Terminate’ IF EFLAG=0 ‘INELIGIBLE’]

[Thank and Terminate]

We’re sorry, but we are looking for people with different medical backgrounds for this study. However, thank you for your interest in this study and for taking the time to answer our questions today.

[IF EFLAG=0 ‘INELIGIBLE, END HERE]

[----------------------------------------New screen----------------------------------------]

[Consent Screen 1]

Consent to Participate

- Please read the following information carefully and select whether you agree to participate or not. Please click here to print a copy of this consent form for your records. [Add link to allow participant to print a copy of consent form.]

How long will this survey take?

- This survey will take approximately 20 minutes to complete.

What information do I need to share to be in this study?

- You do not have to share any information that identifies who you are to participate.

What rights do I have as a study participant?

- Taking part in this study is up to you. You may decide to stop at any time, and you do not have to give a reason for stopping.

What are the benefits to participating?

- There is no guarantee of a direct benefit to you for being in this study. However, the information you provide will help the Crohn’s & Colitis Foundation better serve IBD patients in the future.

What are the risks to participating?

- There are minimal risks to participating in the survey. For example, some questions may make you feel uncomfortable.

Will my responses be shared with others?

- No, your responses will not be shared with others and will be used only for this research.
- Many precautions have been taken to protect your information. The findings from this study will be reported in summary form so that the participants cannot be identified.

[----------------------------------------New screen----------------------------------------]

[Consent Screen 2]

[MANDATORY QUESTION]

[radio; single punch]

CONSENT. If you have read the previous screens and agree to participate, please click ‘Yes’. If not, click ‘No’.

1 Yes, I agree to participate

2 No, I do not agree to participate

[Programmer:

- If Consent = 1 ‘Yes’, GO TO Section B
- If (Consent = 2 ‘No’ or Consent = Null), GO TO REFUSAL SCREEN and terminate survey]

[----------------------------------------New screen----------------------------------------]

[refusal screen]

Thank you for taking the time to answer our questions today.

[IF (CONSENT = 2 ‘No…’ OR CONSENT = NULL), END HERE]

[----------------------------------------New screen----------------------------------------]

[SECTION B. Questions about patient and their disease]

Thank you for taking the time to participate in this survey. The purpose of this survey is to better understand patient experiences and knowledge of Inflammatory Bowel Disease (IBD). IBD includes Crohn’s disease and ulcerative colitis. First, we would like to ask some questions about you and your experience with your IBD.

[OPTIONAL QUESTION]

[NUMERICAL FIELD; INTEGER RANGE 1930 – 2022]

B1. When were you first told by a doctor or other healthcare professional that you had IBD? Please enter four-digit year below. If you don’t remember the exact year provide an estimate.

Text to enter year

[OPTIONAL QUESTION– “Specify” for Other should be optional too]

[radio; Multiple punch]

B2. Have you had any of the following symptoms **due to IBD** in the past 6 months? Select all that apply.

1 Bowel urgency

2 Frequent bowel movements

3 Loose or watery stools

4 Bloody stools

5 Sores or drainage around your anus or rectum

6 Abdominal pain or cramps

7 Nausea and vomiting

8 Poor appetite

9 Unintentional weight loss

10 Feeling of constantly needing to pass stool

11 Needing to have a bowel movement right after eating

12 Passing mucus in the stool

13 Bloating or feeling very full

14 Passing gas a lot

15 Fatigue or feeling very tired

16 Difficulty sleeping or insomnia

17 Back pain

18 Joint pain or swelling

19 Mouth sores

20 Anxiety, low mood, or depression

21 Dermatological issues or skin problems

22 Other (specify)

23 I have not had any symptoms

[OPTIONAL QUESTION]

[radio; SINGLE punch]

B3. Have you ever had any type of surgery **for your IBD**?

1 Yes

2 No

[IF B3 =1 (YES), THEN ASK B3A]

[OPTIONAL QUESTION]

[radio; SINGLE punch]

B3A. Do you currently have an ostomy? An ostomy is a type of surgery that allows stool to pass through a surgically created opening in the body (a stoma) to discharge bodily waste.

1 Yes

2 No

[IF B3 = 1 (Yes), THEN ASK B3B]

[MANDATORY QUESTION]

[radio; single punch]

B3B. Have you had a colectomy? A colectomy is a type of surgery that removes all of your colon.

1 Yes

2 No

[IF B3 = 1 (Yes) & B3A = 2 (No) & B3B = 2 (No), THEN ASK B3C]

[MANDATORY QUESTION]

[Textbox]

B3C. What type of surgery did you have for your IBD? Please type your answer in the textbox below _____________________

[OPTIONAL QUESTION – “Specify” for Other should be optional too]

[SHOW ONLY ONE DRUG AT ONE TIME (i.e., ONE ROW OF THE GRID AT A TIME]

[radio; Multiple punch]

B4. Are you currently taking, or have you ever taken, any of the following medications for your IBD?

|  |  | I am currently taking… | I am not taking now, but have taken in the past... | I have never taken... | I don’t know if I have taken… |
| --- | --- | --- | --- | --- | --- |
| B4_1 Corticosteroids**: Generic Medication Name (Brand Names)** | **Budesonide** (Entocort® EC, UCERIS™)  **Methylprednisolone** (A-Methapred®, Depo-Medrol®, Medrol Dosepak®, Solu-Medrol®)  **Prednisolone** (Oraped®, Prelone®, and Pediapred®)  **Prednisone** (Deltasone®) | ◯ | ◯ | ◯ | ◯ |
| B4_2 Aminosalicylates (5-ASAs)**: Generic Medication Name (Brand Names)** | **Balsalazide** (Colazal®, Giazo™),  **Mesalamine** (Apriso™, Asacol® HD, Canasa®, Delzicol™, Lialda™, Pentasa®, Rowasa®, Mesalamine DR 800 mg),  **Olsalazine** (Dipentum®)  **Sulfasalazine** (Azulfidine®) | ◯ | ◯ | ◯ | ◯ |
| B4_3 Immunomodulator**: Generic Medication Name (Brand Names)** | **Azathioprine 6-MP** (Azasan®, Imuran®)  **Cyclosporine** (Gengraf®, Neoral®, Sandimmune®)  **Mercaptopurine 6-MP** (Purinethol®)  **Methotrexate** (Rheumatrex®)  **Tacrolimus** (Prograf®) | ◯ | ◯ | ◯ | ◯ |
| B4_4 Antibiotics**: Generic Medication Name (Brand Names)** | **Ciprofloxacin** (Cipro®, Proquin®)  **Metronidazole** (Flagyl®) | ◯ | ◯ | ◯ | ◯ |
| B4_5 Biologics or Biosimilars**: Generic Medication Name (Brand Names)** | **Adalimumab** [including biosimilars] (Humira®, Cyltezo™, Hyrimoz™, Abrilada™, Amjevita®, HADLIMA, Hulio®)  **Certolizumab pegol** (Cimzia®)  **Golimumab** (Simponi®)  **Infliximab** [including biosimilars and unbranded] (Remicade®, Renflexis®, Avsola™, Inflectra™, IXIFI™)  **Natalizumab** (Tysabri®)  **Risankizumab** (Skyrizi®)  **Ustekinumab** (Stelara®)  **Vedolizumab** (Entyvio®) | ◯ | ◯ | ◯ | ◯ |
| B4_6 Targeted Synthetic Small Molecules**: Generic Medication Name (Brand Names)** | **Ozanimod** (Zeposia®)  **Tofacitinib** (Xeljanz®)  **Upadacitinib** (RINVOQ®) | ◯ | ◯ | ◯ | ◯ |
| B4_7 Complementary Therapies**: Generic Medication Name (Brand Names)** | **Mind-Body Therapies**  **Vitamins, Minerals, Supplements**  **Probiotics and Microorganisms**  **Medical Cannabis** | ◯ | ◯ | ◯ | ◯ |
| B4_9 Other Medications Not Previously Mentioned | [specify] | ◯ | ◯ |  |  |

[OPTIONAL QUESTION]

[radio; single punch]

B5. Have you had to go to an **emergency room** in the **past 6 months** because of your IBD?

1 Yes

2 No

[OPTIONAL QUESTION]

[radio; single punch]

B5A. Have you had to **stay overnight in the hospital** in the **past 6 months** due to your IBD?

1 Yes

2 No

[----------------------------------------New screen----------------------------------------]

[SECTION C. Questions about patient knowledge of remission and barriers to understanding]

Thank you for answering questions about your experience with IBD. In this section, we want to discuss remission related to IBD (Crohn’s disease or ulcerative colitis).

[OPTIONAL QUESTION]

[radio; MULTIPLE punch – “Specify” for Other should be optional too]

C1. Which of the following statements describes what remission related to IBD means to you? Select all that apply.

1 I feel so well that I no longer need to take any medications for my disease

2 I can reduce the dose or frequency of medications

3 My disease is no longer progressing or worsening

4 My symptoms are reduced (e.g., less pain, bowel urgency, fatigue)

5 I am no longer experiencing any symptoms (e.g., no bowel urgency, pain, fatigue)

6 I can carry out everyday activities (e.g., work, social activities)

7 My doctor or other health care professional tells me that my colonoscopy, labs, or scan results show no signs of disease

8 My doctor or other health care professional tells me that my colon/intestine is healed

9. I feel the way I used to feel before I got sick.

10 Other (specify)

11 Not sure

[OPTIONAL QUESTION– “Specify” for Other should be optional too]

[radio; MULTIPLE punch – NOTE: Response options should only match those selected in c1]

C2. Of these statements, which one **best** describes what remission related to IBD means to you? Select only one.

1 I feel so well that I no longer need to take any medications for my disease

2 I can reduce the dose or frequency of medications

3 My disease is no longer progressing or worsening

4 My symptoms are reduced (e.g., less pain, bowel urgency, fatigue)

5 I am no longer experiencing any symptoms (e.g., no bowel urgency, pain, fatigue)

6 I can carry out everyday activities (e.g., work, social activities)

7 My doctor or other health care professional tells me that my colonoscopy, labs, or scan results show no signs of disease

8 My doctor or other health care professional tells me that my colon/intestine is healed

9. I feel the way I used to feel before I got sick.

10 Other (specify)

11 Not sure

[IF C1= 3, THEN go to c3]

[IF C1 != 3, THEN go to c5]

[OPTIONAL QUESTION– “Specify” for Other should be optional too]

[IF A3 = 1 (Crohn’s disease (CD)) & B3 =1 (YES), THEN PHASE C3 AS FOLLOWS]

[radio; single punch]

C3. What is your top concern with disease progression?

1 Having to undergo another surgery

2 My symptoms returning

3 Losing response to current treatment

4 Having to increase treatment dosage

5 Having to take a new treatment

6 Greater risk of cancer in the future

7 Risk of long term side effects from stronger or different medications

8 Other (specify)

[OPTIONAL QUESTION– “Specify” for Other should be optional too]

[IF A3 = 1 (Crohn’s disease (CD)) & B3 = 2 (NO), THEN PHASE C3 AS FOLLOWS]

[radio; single punch]

C3. What is your top concern with disease progression?

1 Having to undergo surgery

2 My symptoms returning

3 Losing response to current treatment

4 Having to increase treatment dosage

5 Having to take a new treatment

6Greater risk of cancer in the future

7 Risk of long term side effects from stronger or different medications

8 Other (specify)

[OPTIONAL QUESTION– “Specify” for Other should be optional too]

[IF A3 = 2 (Ulcerative colitis (UC) AND B3B=1 (Yes Colectomy)), THEN PHRASE C3 AS FOLLOWS]

[radio; single punch]

C3. What is your top concern with disease progression?

1 Having to undergo another surgery

2 My symptoms returning

3 Losing response to current treatment

4 Having to increase treatment dosage

5 Having to take a new treatment

6 Greater risk of cancer in the future

7 Risk of long-term side effects from stronger or different medications

8 Other (specify)

[OPTIONAL QUESTION– “Specify” for Other should be optional too]

[IF A3 = 2 (Ulcerative colitis (UC)) and b3b = 2 (NO colectomy), THEN PHRASE C3 AS FOLLOWS]

[radio; single punch]

C3. What is your top concern with disease progression?

1 Having a colectomy

2 My symptoms returning

3 Losing response to current treatment

4 Having to increase treatment dosage

5 Having to take a new treatment

6 Greater risk of cancer in the future

7 Risk of long-term side effects from stronger or different medications

8 Other (specify)

[OPTIONAL QUESTION– “Specify” for Other should be optional too]

[IF C3 = 2 TheN CoNTINUE TO C4. OTHERWISE CONTINUE TO C5]

[radio; Multiple punch LIMIT (3)]

C4. What are the top three symptoms you are most concerned will return?

1 Bowel urgency

2 Frequent bowel movements

3 Loose or watery stools

4 Bloody stools

5 Sores or drainage around your anus or rectum

6 Abdominal pain or cramps

7 Nausea and vomiting

8 Poor appetite

9 Unintentional weight loss

10 Feeling of constantly needing to pass stool

11 Needing to have a bowel movement right after eating

12 Passing mucus in the stool

13 Bloating or feeling very full

14 Passing gas a lot

15 Fatigue or feeling very tired

16 Difficulty sleeping or insomnia

17 Back pain

18 Joint pain or swelling

19 Mouth sores

20 Anxiety, low mood, or depression

21 Dermatological issues or skin problems

[OPTIONAL QUESTION]

[radio; single punch]

C5. Do you think remission is possible in IBD?

1 Yes

2 No

3 Not sure

[OPTIONAL QUESTION]

[If c2 != 1 (I feel so well that I no longer need to take any medications for my disease), Then ASK C6]

[radio; single punch]

C6. Would you consider yourself to be in remission if you still had to take medications?

1 Yes

2 No

3 Not sure

[OPTIONAL QUESTION]

[radio; single punch]

C7. Have you **ever discussed remission** with your doctor or another health care professional? Your doctor or health care professional may have used other words to describe remission, such as “no active disease” or “healing of the colon/intestine.”

1 Yes

2 No

3 Not sure or don’t remember

[OPTIONAL QUESTION]

[radio; single punch]

C8. Have you **ever been told you were in remission** by a doctor or other health care professional? Your doctor may have used other words to describe remission, such as “no active disease” or “healing of the colon/intestine.”

1 Yes

2 No

3 Not sure or don’t remember

[IF C8= 2, THEN go to c10]

[IF C8= 3, THEN go to c10]

[OPTIONAL QUESTION– “Specify” for Other should be optional too]

[radio; single punch]

C9. Who told you that you were in remission?

1 Primary care doctor (e.g., internist, family medicine doctor)

2 Gastroenterologist (a doctor who specializes in the digestive system)

3 Nurse Practitioner or Physician Assistant

4 Fellow or resident (i.e., physician undergoing period of medical training)

5 Other (specify)

6 Not sure or don’t remember

[OPTIONAL QUESTION]

[radio; single punch]

C10. How important is it for you to have a discussion about remission with your doctor or health care professional during each of the time points listed below? Please answer on a scale of 1(not at all important) to 5 (very important).

|  | 1  Not at all important | 2 | 3  Somewhat important | 4 | 5  Very important |
| --- | --- | --- | --- | --- | --- |
| C10_1 When I am first told I have IBD | ◯ | ◯ | ◯ | ◯ | ◯ |
| C10_2 After my symptoms are under control | ◯ | ◯ | ◯ | ◯ | ◯ |
| C10_3 During each visit with my doctor or health care professional | ◯ | ◯ | ◯ | ◯ | ◯ |
| C10_4 During flare-ups | ◯ | ◯ | ◯ | ◯ | ◯ |
| C10_5 After a colonoscopy | ◯ | ◯ | ◯ | ◯ | ◯ |
| C10_6 After getting test or lab results | ◯ | ◯ | ◯ | ◯ | ◯ |

[OPTIONAL QUESTION– “Specify” for Other should be optional too]

[radio; MUltiple punch (Limit 2)]

C11. In addition to talking to your doctor or other health care professional, how would you like to receive information about remission related to IBD? Select your two most preferred methods of receiving information.

1 Written material from my doctor that I could take home with me

2 Information on a website that I can trust

3 A video I can watch online

4 From other patients with IBD

5 Other (specify)

[OPTIONAL QUESTION]

[radio; single punch]

For these questions, please think about the doctor or healthcare professional who you have mainly seen over the past year for care related to your IBD.

C12. In the **past year**, how much did your doctors and other health care professionals make you feel comfortable asking questions?

1 Not at all

2 Not very much

3 Somewhat

4 A lot

5 A great deal

[OPTIONAL QUESTION]

[radio; single punch]

C13. In the **past year**, how often did you feel that your doctors and other health care professionals had open and honest communication with you?

1 Never

2 Rarely

3 Sometimes

4 Often

5 Always

[OPTIONAL QUESTION]

[radio; single punch]

C14. Many decisions need to be made in IBD care such as decisions about treatment choices, where to go for care, or how to manage side effects. Please think about all of the decisions there have been in your care over the **past year**.

In the **past year**, how much did your doctors and other health care professionals give you information and resources to help you make decisions?

1 Not at all

2 Not very much

3 Somewhat

4 A lot

5 A great deal

6 Does not apply

[OPTIONAL QUESTION]

[radio; single punch]

C15. In the **past year**, how well did your doctors and other healthcare professionals talk with you about how to cope with any fears, stress, and other feelings related to living with IBD?

1 Poorly

2 Not very well

3 Fairly well

4 Very well

5 Outstanding

6 Does not apply

[OPTIONAL QUESTION]

[radio; single punch]

C16. In the **past year**, how often did your doctors and other health care professionals make sure you understood the steps in your care?

1 Never

2 Rarely

3 Sometimes

4 Often

5 Always

[OPTIONAL QUESTION]

[radio; single punch]

C17. Patients with IBD often face uncertainties about their condition. For example, patients may not know what will happen, how treatment is working, and how to make sense of different information and opinions.

In the **past year**, how well did your doctors and other health professionals help you deal with the uncertainties about IBD?

1 Poorly

2 Not very well

3 Fairly well

4 Very well

5 Outstanding

6 Does not apply or there has not been any uncertainties

[----------------------------------------New screen----------------------------------------]

[SECTION D. Information treatmentS]

We previously asked what remission means to you and whether you have discussed remission with a doctor or other health care professional. On the next screen, we will give you more information on what remission means. Please read this information carefully.

[after the introductory text above, show the respondent one of three information treatments below (D1, D2, or d3). randomize which information treatment they see. ]

[----------------------------------------New screen---------------------------------------]

[Section D1. Information Treatment 1 (control) – Foundation website text ([Partnering with Your Doctor | Crohn's & Colitis Foundation (crohnscolitisfoundation.org)](https://www.crohnscolitisfoundation.org/effective-partnering))]

**[MAKE RESPONDENT WAIT 30 SECONDS ON THIS SCREEN TO ENCOURAGE THEM TO READ THE TEXT]**

Remission is a term that many people with IBD think of as “the absence of symptoms.” However, remission can take different forms, depending on what is being observed:

- ***Symptom control/clinical remission*** is the resolution of active IBD symptoms. This type of remission may come with a higher long-term risk of flaring and treatment failure compared with endoscopic or deep remission described below. This is because the treatments are focused on relieving your current symptoms, which can be helpful for patients to feel better. Other types of remission may target healing the damage and inflammation caused by your disease, as noted below.
- ***Mucosal healing*** (also known as endoscopic remission) refers to an absence of active disease seen during a colonoscopy (e.g., no ulcers, no bleeding). Aiming for mucosal healing lowers your risk of complications such as [**strictures**](https://www.crohnscolitisfoundation.org/what-is-crohns-disease/treatment/surgery/strictureplasty) and [**fistulas**](https://www.crohnscolitisfoundation.org/what-is-crohns-disease/treatment/surgery/fistula-removal).
- ***Histologic remission*** (also known as deep remission) occurs when no active inflammation is seen at the tissue level (when biopsies taken during a colonoscopy are examined under the microscope).

Your healthcare professional(s) will prescribe treatments or in some cases recommend surgery to reach these remission goals.

[SECTION D2. INFORMATION TREATMENT 2 – Foundation website content but with Targeted language to align more with draft updated fda guidelines]

**[MAKE RESPONDENT WAIT 30 SECONDS ON THIS SCREEN TO ENCOURAGE THEM TO READ THE TEXT]**

The term “remission” is used to mean different things in IBD.

- ***Clinical remission*** means that IBD symptoms (e.g., bloody stool, bowel urgency, pain, and fatigue) are under control.
- ***Endoscopic Remission*** means there is no active disease seen during a colonoscopy (e.g., no ulcers, no bleeding).
- ***Histologic remission*** means there is no active inflammation seen at the tissue level. Doctors determine if a patient is in histologic remission by taking a biopsy during a colonoscopy and examining the tissue under a microscope.

A patient that achieves clinical remission may feel better because their symptoms are under control. However, a patient may experience no symptoms and still have inflammation in their digestive system, which increases their risk of adverse outcomes in the future. Patients that reach histologic remission will have their symptoms under control and also have:

- lower risk of flare ups,
- lower risk of complications like [strictures](https://www.crohnscolitisfoundation.org/what-is-crohns-disease/treatment/surgery/strictureplasty) and [fistulas](https://www.crohnscolitisfoundation.org/what-is-crohns-disease/treatment/surgery/fistula-removal), and
- lower risk of developing cancer of the colon or rectum in the future.

You can work with your doctor or other healthcare professional to identify treatments that will help you reach your remission goals.

[SECTION D3. INFORMATION TREATMENT 3 – discussion of short-term and long-term treatment goals with single remission definition]

**[MAKE RESPONDENT WAIT 30 SECONDS ON THIS SCREEN TO ENCOURAGE THEM TO READ THE TEXT]**

**Short-term treatment goal: Control of Symptoms**

The short-term treatment goal of most patients is to get their symptoms under control (e.g., bloody stool, bowel urgency, pain, and fatigue under control). However, a patient may experience no symptoms and still have inflammation in their digestive system, which increases their risk of adverse outcomes in the future.

**Long-term treatment goal: Remission**

The long-term goal of treatment is remission. When patients are in remission, their symptoms are under control and there is no evidence of active disease (e.g., ulcers or bleeding) and possibly no active inflammation at the tissue level.

Doctors determine if there is active disease by looking for ulcers or bleeding during a colonoscopy and by looking for signs of inflammation on scans. Doctors may determine if there is active inflammation at the tissue level by taking a biopsy during a colonoscopy and examining the tissue under a microscope.

Reaching remission has important benefits. Specifically, patients that reach remission will have their symptoms under control and also have:

- lower risk of flare ups,
- lower risk of complications like [strictures](https://www.crohnscolitisfoundation.org/what-is-crohns-disease/treatment/surgery/strictureplasty), [fistulas](https://www.crohnscolitisfoundation.org/what-is-crohns-disease/treatment/surgery/fistula-removal), and
- lower risk of developing cancer of the colon or rectum in the future.

You can work with your doctor or other healthcare professional to identify treatments that will help you reach your treatment goals.

[MANDATORY QUESTION]

D1. Think about the information you just read. How easy or difficult was it to understand?

1 Very easy to understand

2 Someway easy to understand

3 Neither easy nor difficult to understand

4 Somewhat difficult to understand

5 Very difficult to understand

[----------------------------------------New screen----------------------------------------]

[SECTION E. Questions about willingness to undertake medical procedures]

[MANDATORY QUESTION]

[IF Respondent received Information treatment 1 or information treatment 2]

[radio; MULTIPLE punch]

E1A. As far as you are aware, which of the following are long-term benefits of reaching **endoscopic remission**? Select all that apply

1 Symptom control [correct]

2 Lower risk of high blood pressure [incorrect]

3 Lower risk of flare ups [correct]

4 Lower risk of complications like strictures and fistulas [correct]

5 None of the above [incorrect]

[MANDATORY QUESTION]

[IF Respondent received Information treatment 3]

[radio; single punch]

E1B. As far as you are aware, which of the following are long-term benefits of reaching remission?

1 Symptom control [correct]

2 Lower risk of high blood pressure [incorrect]

3 Lower risk of flare ups [correct]

4 Lower risk of complications like strictures and fistulas [correct]

5 None of the above [incorrect]

[MANDATORY QUESTION]

[radio; single punch]

E2. After reading about remission earlier in this survey, do you feel more or less prepared to do the following? In answering the questions, please think about treatment decisions that could help you reach remission. Please answer on a scale of 1 (much less prepared) to 5 (much more prepared).

|  | 1  Much less prepared | 2 | 3  No more or less prepared | 4 | 5  Much more prepared |
| --- | --- | --- | --- | --- | --- |
| E2_1 Discussing remission with my doctor or other health care professional | ◯ | ◯ | ◯ | ◯ | ◯ |
| E2_2 Discussing treatment goals with my doctor or other health care professional | ◯ | ◯ | ◯ | ◯ | ◯ |
| E2_3 Making decisions about treatment that can help me achieve remission | ◯ | ◯ | ◯ | ◯ | ◯ |

[MANDATORY QUESTION]

[radio; single punch]

E3. Treatment makes it more likely that you will go into remission and stay there for an extended period of time. You also may need more frequent doctor’s visits, colonoscopies, and other tests until you achieve remission. How willing would you be to do each of the following to achieve and stay in remission? Please answer on a scale of 1 (not at all willing) to 5 (very willing).

| Statement | 1  Not at all willing | 2  A little  willing | 3  Somewhat willing | 4  Willing | 5  Very willing |
| --- | --- | --- | --- | --- | --- |
| E3_1 Continue use of medications to stay in remission | ◯ | ◯ | ◯ | ◯ | ◯ |
| E3_2 Have an additional colonoscopy | ◯ | ◯ | ◯ | ◯ | ◯ |
| E3_3 Have more frequent lab and other tests | ◯ | ◯ | ◯ | ◯ | ◯ |

[MANDATORY QUESTION]

E4. Finally, please remind us: Which of the following inflammatory bowel disease (IBD) conditions were you most recently told by a doctor or other healthcare professional that you had?

1 Crohn’s disease (CD)

2 Ulcerative colitis (UC)

3 Indeterminate Colitis or Unclassified IBD (IBD-U)

5 Other

6 Prefer not to answer
