# Supplementary material for: Communicating Information Regarding IBD Remission to Patients: Evidence From a Survey of Adult Patients in the United States
Source: Inflamm Bowel Dis. 2024 Aug 28;31(6):1605–15. doi: 10.1093/ibd/izae201 (PMC12166295; doi:10.1093/ibd/izae201)
Supplement: izae201_suppl_Supplementary_Material [file izae201_suppl_supplementary_material.docx]

**Supplemental Figure S1. Flow Diagram for Survey Response Dataset Construction**


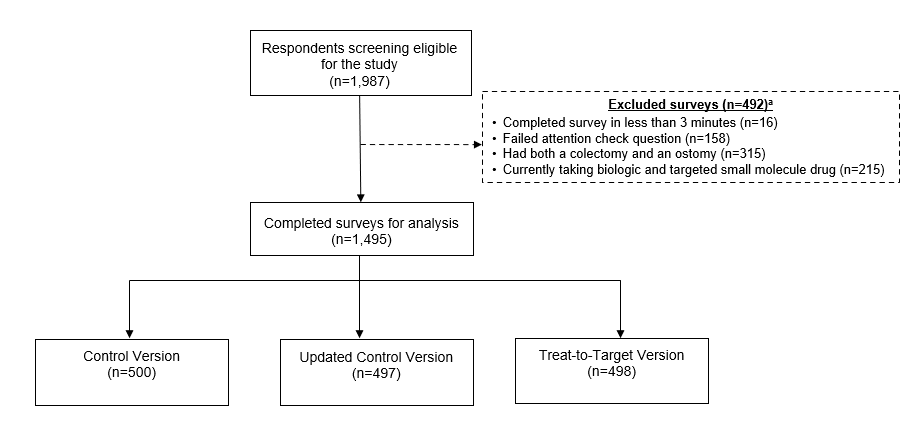


^a^ We excluded 492 respondents for failing at least one quality check. The number of respondents excluded due to each quality check does not add up to 492 because respondents could fail more than one quality check. We excluded 315 respondents who had both a colectomy and an ostomy and 215 respondents who were currently taking a biologic and targeted small molecule drug. Although it is possible for a patient to have both a colectomy and an ostomy or to take both a biologic and targeted small molecule drug, it is very unlikely. Therefore, we excluded this relatively large number of respondents to avoid compromising data quality.

Note: In addition to the pre-identified quality checks discussed above, we also identified 22 respondents that indicated they had all six medical conditions included in the screener question used to determine if the patient had been diagnosed with IBD. Although it seems unlikely that a single individual would have all six medical conditions, we found that our results did not change whether we included these patients or not. Therefore, we chose to include them in the final sample.

**Supplemental Table S1. Patient top concern with disease progression**

|  | Crohn's Disease with surgery (n=111) | Crohn's Disease with no surgery (n=253) | All Crohn's Disease  Patients  (n=364) |
| --- | --- | --- | --- |
| Having to undergo surgery / Having to undergo another surgery | 47.8% | 37.2% | 40.4% |
| My symptoms returning | 18.0% | 16.2% | 16.8% |
| Having to increase treatment dosage | 0.0% | 2.8% | 1.9% |
| Having to take a new treatment | 2.7% | 3.6% | 3.3% |
| Greater risk of cancer in the future | 14.4% | 18.2% | 17.0% |
| Risk of long-term side effects from stronger/different medications | 12.6% | 15.8% | 14.8% |
| Losing response to current treatment | 2.7% | 4.7% | 4.1% |
| Other | 1.8% | 1.6% | 1.6% |
| **Total** | **100.0%** | **100.0%** | **100.0%** |

Note: Data for ulcerative colitis patients excluded due to low response rates for this question.

**Supplemental Table S2. Patient Characteristics by Severity**

| Characteristic | Severe  (IBD Constantly or Often Active in Past 6 Months) (n=758) | Not Severe  (IBD Sometimes, Occasionally, Rarely or Not Active in Past 6 Months) (n=737) | Total (n=1,495) |
| --- | --- | --- | --- |
| **Age, %** (*p*<0.01) |  |  |  |
| 18–24 | 5.4 | 5.2 | 5.3 |
| 25–44 | 64.1 | 39.9 | 52.2 |
| 45–64 | 24.8 | 34.1 | 29.4 |
| 65+ | 5.9 | 20.6 | 13.2 |
| **Gender, %** (*p*<0.01) |  |  |  |
| Female | 57.4 | 68.8 | 63.0 |
| Male | 42.2 | 30.4 | 36.4 |
| Non-binary/Other | 0.4 | 0.8 | 0.6 |
| **Race / Ethnicity, %** (*p*<0.01) |  |  |  |
| White, non-Hispanic | 69.9 | 80.6 | 75.2 |
| Black, non-Hispanic | 6.1 | 5.6 | 5.8 |
| Asian, non-Hispanic | 0.8 | 1.5 | 1.1 |
| Other, non-Hispanic | 5.4 | 2.7 | 4.1 |
| Hispanic | 16.9 | 8.7 | 12.8 |
| Prefer not to answer | 0.9 | 0.9 | 0.9 |
| **Education, %** (*p*=0.78) |  |  |  |
| High school graduate or less | 18.2 | 17.6 | 17.9 |
| Some college or 2-year degree | 34.7 | 35.4 | 35.1 |
| 4-year degree or more | 47.0 | 47.0 | 47.0 |
| Prefer not to answer | 0.0 | 0.1 | 0.1 |
| **Years since patient’s IBD was first diagnosed, %** (*p*<0.01) |  |  |  |
| 1–2 years | 21.8 | 9.4 | 15.7 |
| 3–5 years | 25.1 | 17.8 | 21.5 |
| 6–10 years | 17.7 | 17.6 | 17.7 |
| More than 10 years | 35.5 | 55.2 | 45.2 |
| **Healthcare professional treating patient’s IBD, %** (*p*<0.01) |  |  |  |
| Gastroenterologist | 77.3 | 79.9 | 78.6 |
| Primary care doctor | 20.3 | 15.1 | 17.7 |
| Nurse practitioner or physician assistant | 0.8 | 2.2 | 1.5 |
| Fellow or resident | 0.3 | 0.5 | 0.4 |
| Other specialist | 1.2 | 0.8 | 1.0 |
| Not sure or don’t remember | 1.5 | 0.1 | 0.8 |
| **Total** | **100** | **100** | **100** |

Note: P-values obtained from Chi-square test for the differences in responses across IBD subgroups.

**Supplemental Table S3. Statements Patients Identified as Best Description of What Remission Related to IBD Means to Them by Severity**

| Statement | Severe  (IBD Constantly or Often Active in Past 6 Months) (n=758) | Not Severe  (IBD Sometimes, Occasionally, Rarely or Not Active in Past 6 Months) (n=737) | Total (n=1,495) |
| --- | --- | --- | --- |
| My symptoms are reduced (e.g., less pain, bowel urgency, fatigue). | 22.3 | 21.2 | 21.7% |
| I am no longer experiencing any symptoms (e.g., no bowel urgency, pain, fatigue). | 13.2 | 14.1 | 13.7% |
| I can carry out everyday activities (e.g., work, social activities). | 12.3 | 14.4 | 12.8% |
| My disease is no longer progressing or worsening. | 10.7 | 12.2 | 11.4% |
| I feel so well that I no longer need to take any medications for my disease. | 6.9 | 13.2 | 10.0% |
| My doctor or other healthcare professional tells me that my colonoscopy, labs, or scan results show no signs of disease. | 8.4 | 11.1 | 9.8% |
| I can reduce the dose or frequency of medications | 9.2 | 4.9 | 7.1% |
| I feel the way I used to feel before I got sick. | 6.9 | 3.8 | 5.4% |
| My doctor or other healthcare professional tells me that my colon/intestine is healed. | 2.2 | 1.5 | 1.9% |
| Not sure | 5 | 1.5 | 3.6% |
| Other | 4 | 1.4 | 2.7% |
| **Total** | **100.0%** | **100.0%** | **100.0%** |

Test for differences in responses across disease severity subgroups using Chi-square test (*p* < 0.01).

**Supplemental Table S4. Patient Understanding of Inflammatory Bowel Disease Remission by Severity**

|  | Severe  (IBD Constantly or Often Active in Past 6 Months) (n=758) | Not Severe  (IBD Sometimes, Occasionally, Rarely or Not Active in Past 6 Months) (n=737) | Total (n=1,495) |
| --- | --- | --- | --- |
| IBD remission is possible, % (*p*<0.01) | 62.1 | 71.2 | 66.6 |
| Would consider self to be in remission if still on medication, % (*p*<0.01) | 50.0 | 48.2 | 49.1 |
| Ever discussed remission with doctor, % (*p*<0.01) | 64.2 | 58.6 | 61.5 |
| Ever been told was in remission, % (*p*<0.01) | 34.3 | 42.3 | 38.3 |

Note: P-values obtained from Chi-square test for the differences in responses across disease severity subgroups.

**Supplemental Table S5. Patient Communication Preferences and Experiences by Severity**

| Communication Preference/Experience | Severe  (IBD Constantly or Often Active in Past 6 Months) (n=758) | Not Severe  (IBD Sometimes, Occasionally, Rarely or Not Active in Past 6 Months) (n=737) | Total (n=1,495) |
| --- | --- | --- | --- |
| **Percentage of Patients Selecting a Form of Communication As One of Two Most Preferred Ways of Receiving Information on Remission** |  |  |  |
| Written material from my doctor that I could take home with me, % (*p*=0.06) | 76.5 | 80.5 | 78.5 |
| Information on a website that I can trust, % (*p*<0.01) | 70.3 | 77.9 | 74.1 |
| A video I can watch online, % (*p*<0.01) | 29.7 | 18.2 | 24.0 |
| From other patients with IBD, % (*p*=0.87) | 22.0 | 22.4 | 22.2 |
| Other, % (*p*=0.53) | 1.5 | 1.0 | 1.3 |
| **Percentage of Patients Saying It Is Important or Very Important to Discuss Remission with Your Healthcare Professional at Each Point in Time** |  |  |  |
| When I am first told I have IBD, % (*p*<0.01) | 74.7 | 60.8 | 67.8 |
| After my symptoms are under control, % (*p*=0.03) | 79.6 | 74.0 | 77.3 |
| During each visit with my doctor or healthcare professional, % (*p*<0.01) | 66.5 | 57.7 | 62.1 |
| During flare-ups, % (*p*<0.01) | 67.8 | 58.8 | 63.3 |
| After a colonoscopy, % (*p*=0.41) | 76.5 | 78.3 | 77.4 |
| After getting test or lab results, % (*p*=0.71) | 75.7 | 74.9 | 75.3 |
| **Percentage of Patients Agreeing with Statement About Communicating with Healthcare Professional Over Past Year** |  |  |  |
| Makes me feel comfortable, % (*p*=0.19) | 71.9 | 74.9 | 73.4 |
| Communicates honestly, % (*p*=0.14) | 76.9 | 80.1 | 78.5 |
| Provides information, % (*p*<0.01) | 67.7 | 56.7 | 62.3 |
| Helps me cope with stress from IBD, % (*p*<0.01) | 59.6 | 43.8 | 51.8 |
| Helps me understand steps involved in care, % (*p*=0.13) | 64.3 | 68.0 | 66.1 |
| Helps me deal with uncertainties, % (*p*<0.01) | 57.3 | 47.9 | 52.6 |

Note: P-values obtained from Chi-square test for the differences in responses across disease severity subgroups.

**Supplemental Table S6. Patient Characteristics by Disease Duration**

| Characteristic | Duration Over 10 Years (n=676) | Duration 10 years or less (n=819) | Total (n=1,495) |
| --- | --- | --- | --- |
| **Age, %** (*p*<0.01) |  |  |  |
| 18–24 | 1.2 | 8.7 | 5.3 |
| 25–44 | 40.1 | 62.2 | 52.2 |
| 45–64 | 39.4 | 21.1 | 29.4 |
| 65+ | 19.4 | 8.1 | 13.2 |
| **Gender, %** (*p*<0.01) |  |  |  |
| Female | 72.0 | 55.6 | 63.0 |
| Male | 27.5 | 43.7 | 36.4 |
| Non-binary/Other | 0.4 | 0.7 | 0.6 |
| **Race / Ethnicity, %** (*p*<0.01) |  |  |  |
| White, non-Hispanic | 81.7 | 69.8 | 75.2 |
| Black, non-Hispanic | 5.5 | 6.1 | 5.8 |
| Asian, non-Hispanic | 0.9 | 1.3 | 1.1 |
| Other, non-Hispanic | 4.0 | 4.2 | 4.1 |
| Hispanic | 7.4 | 17.3 | 12.8 |
| Prefer not to answer | 0.6 | 1.2 | 0.9 |
| **Education, %** (*p*<0.01) |  |  |  |
| High school graduate or less | 19.2 | 16.9 | 17.9 |
| Some college or 2-year degree | 38.9 | 31.9 | 35.1 |
| 4-year degree or more | 41.7 | 51.3 | 47.0 |
| Prefer not to answer | 0.2 | 0.0 | 0.1 |
| **Years since patient’s IBD was first diagnosed, %** (*p<*0.01) |  |  |  |
| 1–2 years | 0 | 28.6 | 15.7 |
| 3–5 years | 0 | 39.2 | 21.5 |
| 6–10 years | 0 | 32.2 | 17.7 |
| More than 10 years | 100 | 0 | 45.2 |
| **Healthcare professional treating patient’s IBD, %** (*p*=0.07) |  |  |  |
| Gastroenterologist | 81.4 | 76.3 | 78.6 |
| Primary care doctor | 14.8 | 20.2 | 17.7 |
| Nurse practitioner or physician assistant | 1.5 | 1.5 | 1.5 |
| Fellow or resident | 0.2 | 0.6 | 0.4 |
| Other specialist | 1.2 | 0.9 | 1.0 |
| Not sure or don’t remember | 1.0 | 0.6 | 0.8 |
| **Total** | **100** | **100** | **100** |

Note: P-values obtained from Chi-square test for the differences in responses across IBD disease duration subgroups.

**Supplemental Table S7. Statements Patients Identified as Best Description of What Remission Related to IBD Means to Them by Disease Duration**

| Statement | Duration Over 10 Years (n=676) | Duration 10 years or less (n=819) | Total (n=1,495) |
| --- | --- | --- | --- |
| My symptoms are reduced (e.g., less pain, bowel urgency, fatigue). | 20.0 | 21.2 | 21.7% |
| I am no longer experiencing any symptoms (e.g., no bowel urgency, pain, fatigue). | 16.6 | 11.2 | 13.7% |
| I can carry out everyday activities (e.g., work, social activities). | 11.8 | 13.6 | 12.8% |
| My disease is no longer progressing or worsening. | 12.3 | 10.7 | 11.4% |
| I feel so well that I no longer need to take any medications for my disease. | 13.0 | 7.3 | 10.0% |
| My doctor or other healthcare professional tells me that my colonoscopy, labs, or scan results show no signs of disease. | 9.6 | 9.9 | 9.8% |
| I can reduce the dose or frequency of medications | 4.3 | 9.4 | 7.1% |
| I feel the way I used to feel before I got sick. | 4 | 6.5 | 5.4% |
| My doctor or other healthcare professional tells me that my colon/intestine is healed. | 1.3 | 2.3 | 1.9% |
| Not sure | 3.4 | 3.8 | 3.6% |
| Other | 3.7 | 2 | 2.7% |
| **Total** | **100.0%** | **100.0%** | **100.0%** |

Test for differences in responses across disease duration subgroups using Chi-square test (*p* < 0.01).

**Supplemental Table S8. Patient Understanding of Inflammatory Bowel Disease Remission by Disease Duration**

|  | Duration Over 10 Years (n=676) | Duration 10 years or less (n=819) | Total (n=1,495) |
| --- | --- | --- | --- |
| IBD remission is possible, %  (*p*=0.94) | 66.9 | 66.4 | 66.6 |
| Would consider self to be in remission if still on medication, % (*p*=<0.01) | 45.7 | 51.9 | 49.1 |
| Ever discussed remission with doctor, % (*p*=0.04) | 60.8 | 62.0 | 61.5 |
| Ever been told was in remission, % (*p*=<0.01) | 43.9 | 33.6 | 38.3 |

Note: P-values obtained from Chi-square test for the differences in responses across IBD disease duration subgroups.

**Supplemental Table S9. Patient Communication Preferences and Experiences by Disease Duration**

| Communication Preference/Experience | Duration Over 10 Years (n=676) | Duration 10 years or less (n=819) | Total (n=1,495) |
| --- | --- | --- | --- |
| **Percentage of Patients Selecting a Form of Communication As One of Two Most Preferred Ways of Receiving Information on Remission** | | | |
| Written material from my doctor that I could take home with me, % (*p<*0.01) | 81.8 | 75.7 | 78.5 |
| Information on a website that I can trust, % (*p*<0.01) | 79.1 | 69.8 | 74.1 |
| A video I can watch online, % (*p*<0.01) | 17.9 | 29.1 | 24.0 |
| From other patients with IBD, % (*p*=0.02) | 19.4 | 24.5 | 22.2 |
| Other, % (*p*=0.1) | 1.8 | 0.9 | 1.3 |
| **Percentage of Patients Saying It Is Important or Very Important to Discuss Remission with Your Healthcare Professional at Each Point in Time** | | | |
| When I am first told I have IBD, % (*p*<0.01) | 63.8 | 71.2 | 67.8 |
| After my symptoms are under control, % (*p*<0.01) | 73.5 | 80.3 | 77.3 |
| During each visit with my doctor or healthcare professional, % (*p*=0.02) | 58.9 | 64.8 | 62.1 |
| During flare-ups, % (*p*=0.81) | 63.0 | 63.6 | 63.3 |
| After a colonoscopy, % (*p*=0.82) | 77.7 | 77.1 | 77.4 |
| After getting test or lab results, % (*p*=0.04) | 72.8 | 77.4 | 75.3 |
| **Percentage of Patients Agreeing with Statement About Communicating with Healthcare Professional Over Past Year** |  |  |  |
| Makes me feel comfortable, % (*p*=0.90) | 73.2 | 73.5 | 73.4 |
| Communicates honestly, % (*p*=0.84) | 78.7 | 78.3 | 78.5 |
| Provides information, % (*p*<0.01) | 55.3 | 68.0 | 62.3 |
| Helps me cope with stress from IBD, % (*p*<0.01) | 42.6 | 59.5 | 51.8 |
| Helps me understand steps involved in care, % (*p*=0.34) | 67.2 | 64.8 | 66.1 |
| Helps me deal with uncertainties, % (*p*<0.01) | 45.3 | 58.7 | 52.6 |

Note: P-values obtained from Chi-square test for the differences in responses across disease duration subgroups.

**Supplemental Table S10. Patient Characteristics by Age**

| Characteristic | Respondent Over 40 Years Old (n=778) | Respondent 40 Years Old or Younger (n=717) | Total (n=1,495) |
| --- | --- | --- | --- |
| **Age, %** (*p*<0.01) |  |  |  |
| 18–24 | 0 | 11.0 | 5.3 |
| 25–44 | 18.3 | 89.0 | 52.2 |
| 45–64 | 56.4 | 0.0 | 29.4 |
| 65+ | 25.3 | 0.0 | 13.2 |
| **Gender, %** (*p*<0.01) |  |  |  |
| Female | 70.8 | 54.5 | 63.0 |
| Male | 29.1 | 44.4 | 36.4 |
| Non-binary/Other | 0.1 | 1.1 | 0.6 |
| **Race / Ethnicity, %** (*p*<0.01) |  |  |  |
| White, non-Hispanic | 79.7 | 70.3 | 75.2 |
| Black, non-Hispanic | 6.2 | 5.4 | 5.8 |
| Asian, non-Hispanic | 1.0 | 1.3 | 1.1 |
| Other, non-Hispanic | 4.0 | 4.2 | 4.1 |
| Hispanic | 8.4 | 17.7 | 12.8 |
| Prefer not to answer | 0.8 | 1.1 | 0.9 |
| **Education, %** (*p*<0.01) |  |  |  |
| High school graduate or less | 18.5 | 17.3 | 17.9 |
| Some college or 2-year degree | 39.2 | 30.5 | 35.1 |
| 4-year degree or more | 42.2 | 52.2 | 47.0 |
| Prefer not to answer |  |  | 0.1 |
| **Years since patient’s IBD was first diagnosed, %** (*p*<0.01) |  |  |  |
| 1–2 years | 9.6 | 22.2 | 15.7 |
| 3–5 years | 16.6 | 26.8 | 21.5 |
| 6–10 years | 15.2 | 20.4 | 17.7 |
| More than 10 years | 58.6 | 30.7 | 45.2 |
| **Healthcare professional treating patient’s IBD, %** (*p*=0.18) |  |  |  |
| Gastroenterologist | 80.3 | 76.7 | 78.6 |
| Primary care doctor | 15.9 | 19.7 | 17.7 |
| Nurse practitioner or physician assistant | 1.5 | 1.4 | 1.5 |
| Fellow or resident | 0.1 | 0.7 | 0.4 |
| Other specialist | 1.0 | 0.9 | 1.0 |
| Not sure or don’t remember | 1.0 | 0.6 | 0.8 |
| **Total** | **100** | **100** | **100** |

Note: P-values obtained from Chi-square test for the differences in responses across age subgroups.

**Supplemental Table S11. Statements Patients Identified as Best Description of What Remission Related to IBD Means to Them by Age**

| Statement | Respondent Over 40 Years Old (n=778) | Respondent 40 Years Old or Younger (n=717) | Total (n=1,495) |
| --- | --- | --- | --- |
| My symptoms are reduced (e.g., less pain, bowel urgency, fatigue). | 20.3% | 23.3% | 21.7% |
| I am no longer experiencing any symptoms (e.g., no bowel urgency, pain, fatigue). | 14.5% | 12.7% | 13.7% |
| I can carry out everyday activities (e.g., work, social activities). | 12.9% | 12.7% | 12.8% |
| My disease is no longer progressing or worsening. | 11.7% | 11.2% | 11.4% |
| I feel so well that I no longer need to take any medications for my disease. | 9.8% | 10.2% | 10.0% |
| My doctor or other healthcare professional tells me that my colonoscopy, labs, or scan results show no signs of disease. | 10.9 | 8.5 | 9.8% |
| I can reduce the dose or frequency of medications | 5.5 | 8.8 | 7.1% |
| I feel the way I used to feel before I got sick. | 5.5 | 5.2 | 5.4% |
| My doctor or other healthcare professional tells me that my colon/intestine is healed. | 2.1 | 1.7 | 1.9% |
| Not sure | 3.2 | 4.0 | 3.6% |
| Other | 3.6 | 1.8 | 2.7% |
| **Total** | **100.0%** | **100.0%** | **100.0%** |

Test for differences in responses across age subgroups using Chi-square test (*p* = 0.10).

**Supplemental Table S13. Patient Understanding of Inflammatory Bowel Disease Remission by Age**

|  | Respondent Over 40 Years Old (n=778) | Respondent 40 Years Old or Younger (n=717) | Total (n=1,495) |
| --- | --- | --- | --- |
| IBD remission is possible, % (*p*<0.01) | 61.7 | 72.0 | 66.6 |
| Would consider self to be in remission if still on medication, % (*p*<0.01) | 45.4 | 53.1 | 49.1 |
| Ever discussed remission with doctor, % (*p*<0.01) | 56.0 | 67.4 | 61.5 |
| Ever been told was in remission, % (*p*=0.02) | 35.5 | 41.3 | 38.3 |

Note: P-values obtained from Chi-square test for the differences in responses across age subgroups.

**Supplemental Table S14. Patient Communication Preferences and Experiences by Age**

| Communication Preference/Experience | Respondent Over 40 Years Old (n=778) | Respondent 40 Years Old or Younger (n=717) | Total (n=1,495) |
| --- | --- | --- | --- |
| **Percentage of Patients Selecting a Form of Communication As One of Two Most Preferred Ways of Receiving Information on Remission** |  |  |  |
| Written material from my doctor that I could take home with me, % (*p*<0.01) | 81.8 | 74.9 | 78.5 |
| Information on a website that I can trust, % (*p*<0.01) | 79.3 | 68.3 | 74.1 |
| A video I can watch online, % (*p*<0.01) | 18.9 | 24.0 | 24.0 |
| From other patients with IBD, % (*p*<0.01) | 18.3 | 26.5 | 22.2 |
| Other, % (*p*=0.06) | 1.8 | 0.7 | 1.3 |
| **Percentage of Patients Saying It Is Important or Very Important to Discuss Remission with Your Healthcare Professional at Each Point in Time** |  |  |  |
| When I am first told I have IBD, % (*p*=0.23) | 66.5 | 69.3 | 67.8 |
| After my symptoms are under control, % (*p*=0.89) | 77.1 | 77.4 | 77.3 |
| During each visit with my doctor or healthcare professional, % (*p*=0.95) | 62.2 | 62.1 | 62.1 |
| During flare-ups, % (*p*=0.23) | 64.8 | 61.8 | 63.3 |
| After a colonoscopy, % (*p*<0.01) | 80.3 | 74.2 | 77.4 |
| After getting test or lab results, % (*p*<0.01) | 78.2 | 72.3 | 75.3 |
| **Percentage of Patients Agreeing with Statement About Communicating with Healthcare Professional Over Past Year** |  |  |  |
| Makes me feel comfortable, % (*p*=0.19) | 74.8 | 71.8 | 73.4 |
| Communicates honestly, % (*p*=0.34) | 79.4 | 77.4 | 78.5 |
| Provides information, % (*p*<0.01) | 57.1 | 67.9 | 62.3 |
| Helps me cope with stress from IBD, % (*p*<0.01) | 46.5 | 57.6 | 51.8 |
| Helps me understand steps involved in care, % (*p*=0.37) | 65.0 | 67.2 | 66.1 |
| Helps me deal with uncertainties, % (*p*<0.01) | 46.0 | 59.8 | 52.6 |

Note: P-values obtained from Chi-square test for the differences in responses across age subgroups.
